# Congenital Anomalies of the Knee—Hypoplasia of the Meniscus: A Case Report

**DOI:** 10.1155/cro/6636850

**Published:** 2026-05-18

**Authors:** Arianna Paa, Doyle Ford, William Woodhams, William Galardi, Abdoul Mbengue, Micah Lissy

**Affiliations:** ^1^ Department of Orthopedics, Michigan State University College of Human Medicine, East Lansing, Michigan, USA, msu.edu; ^2^ Department of Orthopedics, Michigan State University College of Osteopathic Medicine, East Lansing, Michigan, USA, msu.edu

## Abstract

**Background:**

Congenital anomalies involving the meniscus and anterior cruciate ligament (ACL) are rare and often discovered incidentally. Discoid meniscus is the most frequently reported congenital variant, whereas medial meniscal hypoplasia and ACL agenesis remain exceptionally uncommon.

**Case Presentation:**

A 16‐year‐old male presented with right knee pain, swelling, and instability following a noncontact basketball injury. He reported a similar episode weeks earlier. Clinical examination revealed anterior laxity, and magnetic resonance imaging (MRI) demonstrated a nonvisualized ACL consistent with a tear and absent tissue suggestive of a subacute injury. The medial meniscus appeared diminutive without evidence of a tear or displaced fragment. Arthroscopy confirmed a hypoplastic medial meniscus without reparable tissue and complete absence of ACL fibers. The patient underwent ACL reconstruction with internal brace augmentation and anterolateral ligament (ALL) reconstruction. A lateral meniscal tear was also repaired. Postoperative recovery demonstrated progressive improvement in range of motion and quadriceps activation.

**Conclusion:**

This case highlights a rare presentation of concurrent medial meniscal hypoplasia and congenital absence of the ACL in an otherwise healthy adolescent male. Notably, the patient sustained a pivoting sports‐related injury accompanied by an instability episode, in the absence of any syndromic or systemic congenital anomalies. Recognition of congenital intra‐articular anomalies is essential for accurately interpreting preoperative imaging and understanding that structures such as the ACL and meniscus may not develop in tandem, which can influence surgical planning and clinical decision making.

## 1. Introduction

Congenital anomalies of the knee, such as discoid meniscus, aberrant ligament attachments, meniscal agenesis, and ligament agenesis, are uncommon but increasingly recognized in orthopedic practice [1]. Discoid menisci are the most frequently identified variant, with a prevalence of approximately 4.88 per 100,000 orthopedic patients and are often discovered in the lateral compartment incidentally [2]. Congenital hypoplasia of the meniscus is exceptionally rare, with only a limited number of case reports describing this anomaly [1, 3], whereas current literature suggests that congenital anterior cruciate ligament (ACL) aplasia may occur in approximately 1 in 6000 births [4]. As observed in this case and the current literature, these congenital conditions typically have subclinical courses through adolescence and often become apparent only after trauma, repetitive loading, or advanced imaging [5, 6], posing a significant barrier to estimating the true prevalence of congenital knee anomalies as well as highlighting the diagnostic and therapeutic challenges in identifying and treating these anomalies in clinical practice [1, 2, 4].

Meniscal and cruciate ligament anomalies are believed to result from disruptions in early fetal development. Abnormalities in the composition of synovial fluid leading to impairment of normal fibrocartilage development is one hypothesis for hypoplasia of the meniscus [7]. The menisci, knee capsule, and ligaments form around 4–6 weeks of gestation, with the ACL completely differentiated by Week 8 [8]. Developmental errors around this time may explain the coexistence of meniscal and ligamentous deficiencies, and their range of presentation observed across patients [8, 9]. Reports of bilateral medial and lateral meniscal hypoplasia, as well as ACL agenesis coexisting with discoid meniscus or tibial intercondylar dysplasia, illustrate the spectrum and complexity of these congenital presentations [3, 8].

Diagnosing congenital absence of the meniscus or cruciate ligaments is challenging, as MRI features such as absent fibers, high signal intensity, or abnormal morphology can mimic chronic ligament or meniscal tears [1, 8]. These anomalies are often identified unexpectedly during arthroscopy or reconstructive procedures, requiring intraoperative judgment and adaptation of surgical plans, such as decisions regarding meniscal transplantation or modified ACL reconstruction (ACLR) [3, 5]. However, the clinical relevance of these findings remains unclear, as standardized management guidelines are lacking. They also carry important implications for long‐term joint health, including altered biomechanics, early onset osteoarthritis, and the need for patient counseling regarding activity modification and prognosis [10, 11]. This case adds to the limited literature by presenting the surgical management and clinical course of a rare combination of medial meniscal hypoplasia and congenital absence of the ACL in an otherwise healthy adolescent male [1, 3, 5, 8].

## 2. Consent

This is a single‐patient case report and does not constitute human subjects research as defined by federal regulations, as it does not involve a systematic investigation designed to contribute to generalizable knowledge. Accordingly, Institutional Review Board approval was not required per institutional guidelines; however, written informed consent was obtained from the patient for publication of the clinical details and accompanying images. All identifying information has been removed to protect patient confidentiality. The CARE guidelines were adhered to for the reporting of this case.

## 3. Case Presentation

### 3.1. Injury and Initial Visit

A 16‐year‐old male with no significant medical or surgical history presented with right knee pain, swelling, and instability following a noncontact injury sustained during recreational basketball. While attempting a layup, he reported a sudden giving‐way sensation in the knee, followed by immediate swelling and discomfort. The patient recalled an episode 6 weeks earlier at a trampoline park, during which he landed awkwardly on the same knee and experienced transient swelling and pain. He did not seek medical evaluation at that time and returned to full activity without formal treatment or rehabilitation. On examination, the patient was ambulatory without an assistive device. Inspection of the right knee revealed no erythema or ecchymosis. Ligamentous testing demonstrated anterior instability, with a 2B‐grade anterior drawer and Lachman test. Posterior drawer, varus, and valgus stress testing were stable on examination.

Additionally, McMurray′s and patellar apprehension tests were negative when evaluated, and there was no clinical evidence of meniscal entrapment or patellofemoral instability. The remainder of the musculoskeletal examination was unremarkable, with normal gait mechanics, symmetric lower extremity alignment, and no evidence of joint line tenderness, muscular atrophy, or ligamentous laxity beyond the findings noted in the right knee. The left knee was evaluated clinically for comparison and showed no abnormalities. Thus, the left knee was not clinically or radiologically assessed further.

### 3.2. Radiological Investigations

**Figure 1:**
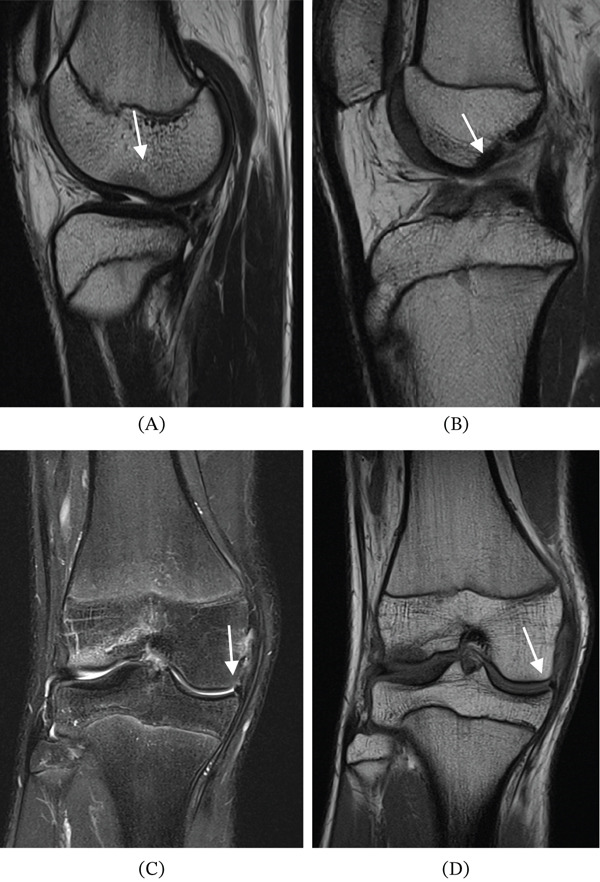
Preoperative MRI demonstrating meniscal morphology and ACL deficiency. Magnetic resonance imaging of the right knee demonstrating (A–B) sagittal views with absence of identifiable anterior cruciate ligament (ACL) fibers within the intercondylar notch and associated bone marrow edema pattern consistent with pivot‐shift injury. (C–D) Coronal and sagittal views illustrating a diminutive medial meniscus with reduced height and width across the anterior horn, midbody, and posterior horn, without evidence of displaced meniscal tear.

Magnetic resonance imaging (MRI) of the right knee was obtained following the patient′s presentation. MRI demonstrated bone marrow edema involving the lateral femoral condyle and posterolateral tibial plateau, consistent with a pivot‐shift injury pattern classically associated with ACL tear. On coronal and sagittal imaging, the ACL was not clearly visualized in its expected anatomical location, with no identifiable intact fibers, raising concern for either a chronic complete rupture with resorption or possible congenital absence as later realized (Figure 1).

Additionally, the medial meniscus appeared diminutive throughout on coronal and sagittal imaging with no displaced meniscal fragment noted. Height and width of the medial meniscus in the anterior horn, midbody, and posterior horn measured below normal range on MRI. Heights for the anterior horn, midbody, and posterior horn of the medial meniscus measured below normal range at 2, 3.3, and 2.8 mm, respectively. Widths of the anterior horn, midbody, and posterior horn of the medial meniscus were also low at 4.6, 3.2, and 3.9 mm. For the lateral meniscus, height measures of the anterior horn and midbody were within normal limits as well as the width of the posterior horn. The width of the anterior horn of the lateral meniscus measured above normal limits at 14 mm, whereas width of the midbody and height of the posterior horn of the lateral meniscus measured below at 5.9 and 3 mm, respectively [7]. A summary of these findings compared to reference measurements can be found in Table 1.

**Table 1:** Participant meniscal characteristics.

Structure	Plane measures	Reference measures (Erbagci et al. 2003)	Patient measures
Medial meniscus			
*Anterior horn*	SH	5.32 ± 0.95	2.00
	CW	7.78 ± 1.86	4.60
*Midbody*	CH	5.03 ± 0.91	3.30
	CW	7.37 ± 2.65	3.20
*Posterior horn*	SH	5.53 ± 0.99	2.80
	CW	11.71 ± 2.63	3.80
Lateral meniscus			
*Anterior horn*	SH	4.33 ± 0.98	3.60
	CW	8.88 ± 2.30	14.00
*Midbody*	CH	4.94 ± 0.99	4.70
	CW	8.37 ± 0.83	5.90
*Posterior horn*	SH	5.36 ± 1.03	3.00
	CW	9.70 ± 41.69	11.00

*Note:* Reference values (mean ± SD) and corresponding patient‐specific measures for medial and lateral meniscal regions in millimeters (mm), including the anterior horn, midbody, and posterior horn. Measurements are presented across imaging planes, including sagittal height (SH), coronal height (CH), and coronal width (CW), to facilitate comparison between normative data and the patient profile.

Notch width index measured in the coronal view was calculated as 0.23. Morphologically, the lateral and medial tibial spines appeared within normal limits without findings of hypoplasia, measuring 10.2 and 10.0 mm, respectively [12]. The intercondylar notch and trochlear groove also demonstrated normal morphology on MRI without findings of trochlear dysplasia.

### 3.3. Decision Making

At the time of presentation, the primary working diagnosis was a chronic ACL rupture with a concurrent medial meniscus injury. This was based on the patient′s history of two distinct traumatic episodes, clinical signs of instability, and a characteristic pivot‐shift bone marrow edema pattern on MRI. The clinical context—particularly the earlier trampoline‐related episode 6 weeks prior—led to the presumption that the absence of clear meniscal and ACL tissue on MRI was a result of chronic degeneration and ACL tear with subsequent resorption of the ligament. At the time, the operative surgeon was unaware of the existence of ACL agenesis and meniscal hypoplasia as entities; with this, the possibility of a congenitally hypoplastic medial meniscus or ACL deficiency was not considered preoperatively. Preoperative planning included expectations of identifying a displaced meniscal tear during arthroscopy, despite its absence on imaging. Based on the clinical and imaging findings, the decision was made to proceed with right knee ACLR using quadriceps tendon autograft with internal bracing and possible anterolateral ligament (ALL) reconstruction with gracilis allograft.

### 3.4. Surgical Intervention

The patient underwent right knee arthroscopy 6 weeks after his initial visit, with the primary goal of ACLR. Examination under anesthesia revealed 3B‐grade anterior drawer and anterior Lachman test, consistent with the patient′s initial physical exam, as well as a 1B‐grade valgus stress test and positive McMurray′s. In addition, a Grade 3 pivot shift was revealed, indicating significant rotatory instability, and as a result, a decision was made intraoperatively to perform an ALL reconstruction using gracilis allograft tissue in addition to the ACLR to lower risk of retear.

**Figure 2:**
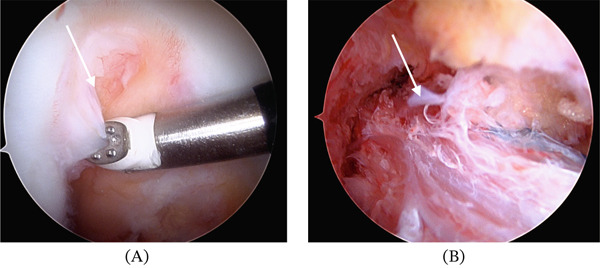
Arthroscopic confirmation of ACL aplasia. Arthroscopic views (A) of the intercondylar notch demonstrating complete absence of native anterior cruciate ligament fibers. Intercondylar notch is narrow but not partially closed. (B) ACLR with internal brace.

Standard anterolateral viewing and anteromedial working portals were established for ACLR. No residual native ACL fibers were observed in the intercondylar notch (Figure 2A). A soft tissue debridement of the intercondylar notch of the femur revealed an Omega notch. An adequate notchplasty was performed to prevent graft impingement. The center of the anatomic footprint of the femoral attachment of the ACL was identified and marked. A 25 × 10 mm femoral tunnel was retrodrilled at an angle 20° from the horizontal and 60° from the anterior condylar line. Next, the tibial footprint was identified and marked, and a 30 × 10 mm tibial tunnel was established. A 7‐cm quadriceps tendon autograph was procured and tensioned to 20 N prior to fixation. Femoral fixation was achieved using an adjustable‐loop suspensory fixation device passed through the medial portal and secured outside the lateral femoral cortex. The graft was tensioned until 15 mm of the graft was visualized entering the femoral bone tunnel. With the knee in full extension, a 14‐mm cortical button was affixed to the adjustable‐loop fixation device. Additional backup fixation was achieved with a 4.75‐mm knotless suture anchor. Suture tape internal brace augmentation was performed followed by final tensioning of the femoral and tibial sides (Figure 2B). Lachman′s test and pivot shift were found to be stable following reconstruction, and arthroscopic evaluation confirmed appropriate size and tension of the graft.

**Figure 3:**
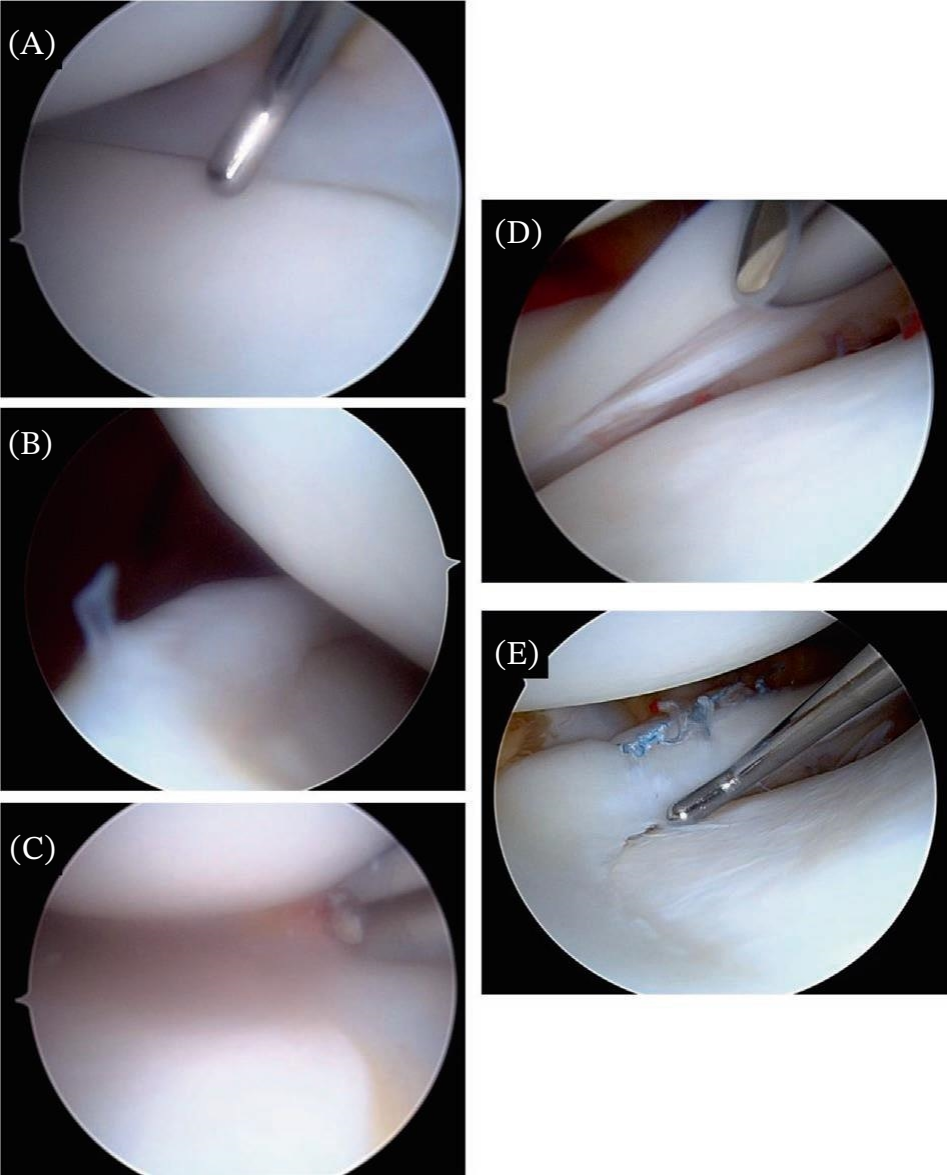
Arthroscopic visualization of meniscal and ligamentous findings. Arthroscopic images demonstrating intraoperative findings within the medial and lateral compartments of the right knee. (A) Hypoplastic medial meniscus with reduced size and no evidence of traumatic pathology. (B) Posterior horn of the medial meniscus demonstrating diminutive morphology. (C) Region of the medial compartment demonstrating relative absence of normal meniscal tissue. (D) Lateral meniscus with a tear identified in the posterior horn. (E) Lateral meniscus following repair using an all‐inside technique.

Intraoperatively, a tear of the posterior horn of the lateral meniscus was identified and repaired with three sutures utilizing an all inside technique (Figure 3).

Based on preoperative clinical and imaging findings, a displaced medial meniscus fragment was anticipated; however, arthroscopy revealed no such fragment and no signs of tearing. Instead, the medial meniscus appeared hypoplastic, with reduced volume, approximately 10% of a normal meniscal thickness, and no reparable tissue (Figure 3) [7]. No surgical intervention was performed for the medial meniscus. Lack of identifiable ACL tissue, along with the hypoplastic appearance of the medial meniscus, raised concern for a possible congenital deficiency rather than purely chronic traumatic changes. These unexpected findings required real‐time adaptation of the surgical approach and contributed to a revised understanding of the patient′s underlying anatomy.

Given the Grade 3 pivot shift revealed on physical examination under anesthesia, an ALL reconstruction was performed with gracilis allograft. For femoral fixation, an incision was made just posterior and proximal to the lateral femoral epicondyle. A guide pin was placed 4 mm posterior and 8 mm proximal to the lateral femoral epicondyle, and the femoral tunnel was established using a 2.7 drill bit. The whipstiched end of the gracilis was passed through the tunnel and secured with a knotless suture anchor. Distally, a second incision was made midway between the fibular head and Gerdy′s tubercle and approximately 1 cm distal to the joint line. A guide pin was placed at the intended tibial attachment site. The graft was passed deep to the iliotibial band and over the guide pin, and isometry was assessed by cycling the knee between 30° and 90° of flexion. After confirming appropriate isometric positioning, a 20 × 7 mm was reamed, and the graft was secured distally with a 7 × 19 mm knotless suture anchor with the knee positioned in 20° of flexion and neutral rotation. The knee was again cycled to confirm that it was not overconstrained.

### 3.5. Outcome and Follow‐Up

**Figure 4:**
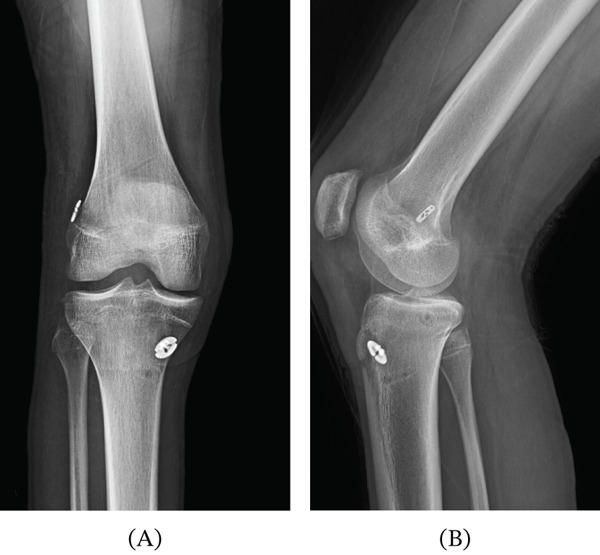
Postoperative radiographic evaluation following ACL and ALL reconstruction. Postoperative anteroposterior and lateral radiographs of the right knee demonstrating appropriate positioning of fixation devices following anterior cruciate ligament reconstruction with internal brace augmentation and anterolateral ligament reconstruction. No evidence of hardware complication or malalignment is observed.

Overall, the patient′s postoperative course and recovery were uncomplicated, and postoperative X‐ray was without abnormality (Figure 4). At 2 weeks postoperatively, the patient presented ambulating with a right knee brace and crutches and reported minimal pain. Physical examination revealed right knee range of motion (ROM) of −5° to 85° and absent quadriceps activation. The patient encountered difficulties accessing consistent physical therapy, which limited early rehabilitation progress. By 8 weeks postoperatively, the patient ambulated to clinic without assistive devices and reported no pain and adherence to physical therapy. The patient demonstrated improved ROM (−3° to 90°). Quadriceps activation was improved but remained mildly diminished. At 12 weeks postoperatively, the patient presented without pain and noted ability to shoot a basketball without discomfort. At this visit, quadriceps activation was markedly improved and comparable to the contralateral limb. The patient was cleared to begin incline running but not yet approved for return to play. The remainder of postoperative course was unremarkable. The patient‐reported outcomes measured at 11 months postoperatively were as follows: 93% International Knee Documentation Committee (IKDC), 68% Knee Injury and Osteoarthritis—Symptoms (KOOS‐Symptoms), 56% KOOS‐Quality of Life subscale, and 55% ACL‐Return to Sports after Injury. At latest follow‐up, approximately 12 months postoperatively, ROM was 0°–140°, and the patient was able to demonstrate a straight leg raise without lag. In addition, the patient was able to squat without pain, with minimal left hip shift. The patient was cleared for return to basketball during this visit with instruction to continue working on strengthening the quadriceps to support stability. To date, the patient has returned to all activities including weightlifting and basketball.

## 4. Discussion

In this case, a hypoplastic medial meniscus and absent ACL were identified intraoperatively in an otherwise healthy adolescent following a traumatic injury. The absence of meniscal and ligamentous fragments, prior surgery or meniscectomy, or high‐impact repetitive trauma strongly suggests a developmental etiology. While incidental findings of meniscal hypoplasia have been reported, the coexistence of this condition with ACL deficiency in a previously asymptomatic patient underscores the incomplete recognition of these anomalies as a distinct clinical entity [1, 3, 5, 6].

Manner et al. [13] established a radiographic classification system for cruciate ligament congenital anomalies as follows: Type I: hypoplastic or aplastic ACL with normal posterior cruciate ligament (PCL) as well as partial closure of the femoral intercondylar notch and hypoplasia of the tibial eminence; Type II: aplastic ACL hypoplastic PCL with aplastic lateral and hypoplastic medial tibial spine forming a “dromedary” sign; and Type III: aplastic ACL and PCL, with bilateral aplasia of the tibial spines and aplasia of the femoral intercondylar notch [6, 13]. While this classification system acts as a foundation to characterizing dysplasia of the cruciate ligaments, it is limited by the fundamental basis of the system being bone abnormalities, namely congenital femoral deficiency and fibular hemimelia, requiring limb lengthening and surgical intervention [13]. Thus, while our patient demonstrated ACL aplasia, the absence of bone abnormalities and any past medical history precludes proper classification with this system. Again, underscoring the complexity of ACL aplasia as its existence in otherwise healthy individuals without syndromic conditions challenges previous understanding of this phenomenon [4, 5, 13].

No formal classification system currently exists for meniscal hypoplasia, but features may include reduced height and width of meniscal tissue, abnormal attachments, and/or abnormal morphology [1, 3, 7].Given this broad definition, our patient does fit criteria for medial meniscal hypoplasia evidenced by not only the diminutive appearance on MRI and arthroscopy but also measurements of width and height throughout the anterior horn, posterior horn, and midbody being below normal limits (Table 1 and (Figure 3A–C).

Regarding the management of congenital anomalies of the knee, the current literature suggests that conservative management with observation only is preferred for asymptomatic or minimally symptomatic patients [4]. Albeit, the relevance of this recommendation may be academic considering that these anomalies are often discovered incidentally when patients are already symptomatic [3, 5, 6]. For symptomatic ACL aplasia, ACLR has proved to be a viable option, demonstrating vast improvements in patient‐reported outcomes and low revision rates at follow‐up. Whether this persists with long‐term follow‐up (> 5 years) remains unclear [4, 14]. Current recommendations for hypoplasia of the menisci are limited, but the case reports detailing management of this anomaly support repair and preservation when possible [1, 15].

In our case, right knee ACLR was performed with no intervention specific to the hypoplastic medial meniscus. At the time of the operation, the surgeon was not aware of congenital anomalies such as medial meniscal hypoplasia due to the rarity of such. With this, it was unclear to the surgeon if meniscus repair would have been beneficial for the patient given that this intraoperative discovery was not the primary cause of the patient′s acute symptoms. Postoperatively, medial meniscus hypoplasia became the hypothesis for the patient′s meniscus abnormality, given that it was significantly diminutive throughout and a displaced meniscal fragment was not recovered (Table 1). With no clear management strategy proving long‐term efficacy for hypoplasia of the meniscus, the patient′s clinical improvement, as well as the risks associated with an additional operation, repair of the medial meniscus was not explored despite this revelation [1, 15]. Regardless of the running hypothesis of congenital hypoplasia, the contralateral knee was not explored through imaging given financial considerations and lack of indication that this would alter future clinical management. While not considered standard of care for ACLR, internal bracing was performed due to evidenced reduction in failure rate; moreover, the operating surgeon performs this with each ACLR regardless of suspected congenital anomaly [16]. Overall, the patient had an uncomplicated postoperative course with no further complaints of pain to date despite intraoperative discoveries complicating surgical management, highlighting the ambiguity in management of congenital hypoplasia of the meniscus.

The case presented raises several gaps in knowledge to be further explored regarding congenital anomalies of the knee, most pressing being effective management strategies in cases of medial meniscus hypoplasia and ACL aplasia. While internal bracing with ACLR was not specific to this patient′s meniscal pathology, early ACLR has been evidenced to preserve the medial meniscus following acute ACL injury [17]. Whether this protective benefit persists in cases of congenital hypoplasia of the medial meniscus remains to be explored. To draw a parallel of this exceptionally rare case, the high incidence of medial meniscus tears in ACL‐deficient knees is well documented, and studies have found that up to 77% of ACL tears are associated with meniscal injury, with medial meniscus involvement more common than lateral [18]. The medial meniscus serves a critical role within sagittal stability and load transmission, and its absence may accelerate joint degeneration or a higher rate of ACLR failure. Chronic ACL deficiency has been linked to post‐traumatic osteoarthritis (PTOA), with some studies reporting a prevalence as high as 87% within these individuals [10, 11]. Whether congenital absence confers the same long‐term risk of PTOA is unclear but raises important considerations for a future study of joint mechanics and future degeneration in this population.

## 5. Conclusion

This case adds to the literature by reporting the surgical management and outcomes of a rare case of concomitant medial meniscus hypoplasia and ACL aplasia. The subclinical course prior to injury in our patient highlights the possibility of unrecognized compensatory mechanisms that may support functional knee stability despite the congenital absence of key stabilizing structures. It is unclear if ACLR in isolation is adequate for stability or if meniscal transplant is required to prevent the increased risk for ACL failure and PTOA. This highlights the importance of recognizing congenital meniscal and ligamentous deficiencies as distinct entities, as they may be present on imaging but overlooked without specific awareness of their features. Historically, these anomalies were often identified intraoperatively due to limited access to advanced imaging; however, with modern MRI, careful preoperative recognition is possible and critical for surgical planning and patient counseling. Importantly, while a congenital anomaly was identified in this case, the patient exhibited no evidence of a syndromic condition or systemic congenital disorder.

Recognizing congenital deficiencies is essential not only for accurate diagnosis but also for setting appropriate patient expectations and guiding postoperative care. Further research is warranted to better define the prevalence, management, and long‐term functional outcomes associated with combined or isolated congenital meniscal and ligamentous deficiencies.

## Funding

No funding was received for this manuscript.

## Conflicts of Interest

The authors declare no conflicts of interest.

## Data Availability

Data sharing not applicable to this article as no datasets were generated or analyzed during the current study.
